# ‘PREMM 1,2,6 MODEL’ as a new gene specific prediction model for Lynch Syndrome: retrospective review of mutation positive cases

**DOI:** 10.1186/1897-4287-10-S2-A67

**Published:** 2012-04-12

**Authors:** J Gale

**Affiliations:** 1 Hereditary Cancer Clinic, Prince of Wales Hospital, High St, Randwick, Sydney, Australia

## 

Lynch Syndrome, also called Hereditary NonPolyposis Colorectal Cancer (HNPCC), occurs as a consequence of germline mutations in the mismatch repair (MMR) system (Stoffel *et al*., 2009), mainly in *MLH1* and *MSH2* (>90% of cases), but also in *MSH6* and *PMS2 *[1].

Kastrinos *et al*., (2011) developed a new web-based multivariable polytomous logistic regression model for Providing gene specific Risk Estimates (Model) (PREMM 1,2,6) that uses genotype-phenotype data from *MLH1*, *MSH2*, and *MSH6* mutation carriers to generate individualized predictions for each gene based on personal and family history characteristics [1] recommends that a gene prediction of >5% should be a threshold for a referral for further genetic risk assessment.

A search was performed to obtain a list of patients seen at the Prince of Wales Hereditary Cancer Clinic who were sequenced for mutations in *MLH1*, *MSH2* or *MSH6* from 1994 to 2011 with 78% occurring after 2005. A total of 40 patient pedigrees were obtained and used for the PREMM 1,2,6 model, 29 were mutation positive and 11 were tested inconclusive. 17% of probands were selected for testing based on tumour testing and the remainder were selected based on family history alone. The model included proband and first and second degree relative’s age and diagnosis of colorectal cancer and endometrial cancer. This model also used an inclusion for extra colonic Lynch-associated cancers. Each proband was also assessed by the Amsterdam II criteria and given a risk category according to the Australian Cancer Network Guidelines of average, 1, 2 or 3.

A gene-specific mutation prediction cut off of 5% was instigative of a mutation for 21 probands (8/12 for *MLH1*, missing 33%, 11/13 for *MSH2*, missing 2% and 2/4 for *MSH6*). Based on this limited data, endometrial cancer was highly predictive for *MSH6*. The prevalence of extracolonic, nonendometrial HNPCC-associated cancers was higher among *MSH2* carriers than *MLH1* and *MSH6* carriers, being weakly predictive. The specific gene estimates were not useful for when tumour analysis was unavailable, with 59% (17) mutation positive probands having a low Premm1,2,6 score, which would not have support a decision to pursue single gene testing. Amsterdam II criteria and Australian Network Guidelines have been previously found to be an ineffective strategy for triage for gene testing for Lynch Syndrome. Further study on a larger cohort is needed to determine if the PREMM 1,2,6 model is an effective triage tool for gene testing for Lynch Syndrome.
